# Treatment of Youth‐Onset Type 2 Diabetes: Focus on SGLT‐2 Inhibitor Use

**DOI:** 10.1155/pedi/4555805

**Published:** 2026-04-01

**Authors:** Silva Arslanian, Timothy Barrett, Naim Shehadeh

**Affiliations:** ^1^ UPMC Children’s Hospital of Pittsburgh, University of Pittsburgh School of Medicine, Pittsburgh, Pennsylvania, USA, pitt.edu; ^2^ University of Birmingham, Birmingham, UK, birmingham.ac.uk; ^3^ Azrieli Faculty of Medicine, Bar-Ilan University, Safed, Israel, biu.ac.il

**Keywords:** adolescents, childhood, pediatric, sodium–glucose co-transporter-2, treatment, type 2 diabetes

## Abstract

The upward trajectory of childhood and adolescent type 2 diabetes (T2D; youth‐onset T2D) is a global health issue, disproportionately affecting non‐white, ethnically diverse, and socioeconomically disadvantaged subgroups. Youth‐onset T2D displays a more aggressive phenotype, with faster disease progression and earlier onset of severe complications when compared to adult‐onset T2D. Despite this, the therapeutic options for the management of youth‐onset T2D have historically been limited. Over the last few years, multiple new therapies across a range of drug classes, including glucagon‐like peptide‐1 receptor agonists (GLP‐1 RAs) and sodium–glucose cotransporter‐2 (SGLT‐2) inhibitors, have been approved for use in youth‐onset T2D. Despite this, patient recruitment to pediatric and adolescent trials remains a major challenge in youth‐onset T2D, and there is currently a lack of large‐scale, long‐term clinical evidence for new treatment classes in this patient population. In this review, we provide an overview of the current treatment landscape in youth‐onset T2D and summarize the available clinical data for SGLT‐2 inhibitors in patients with youth‐onset T2D, based on a targeted literature search.

## 1. Introduction

The global rise in childhood and adolescent obesity has resulted in an increased incidence and prevalence of type 2 diabetes (T2D) in children and adolescents [1–3], with the increase even more pronounced in non‐white, ethnically diverse subgroups [3–5]. In 2021, there were ~41,600 new cases of childhood and adolescent T2D worldwide, with the highest incidence in China, India, and the United States [6]. Between 2002 and 2012, the incidence of T2D in the United States increased by ~4.8% annually, with the greatest increases observed in Native Americans (8.9%), Asian or Pacific Islanders (8.5%), and non‐Hispanic Blacks (6.3%) [7]. These increasing incidence rates resulted in ~28,000 patients with T2D under the age of 20 years in the United States by 2017 [8]. Obesity, increased adiposity, and Native American, Black, Hispanic, or Southern Asian heritage are the major risk factors for youth‐onset T2D, with an estimated 85% of patients in North America either overweight or obese at diagnosis [9, 10]. With the continued rise of obesity and its consequent metabolic derangements, the incidence of T2D in young people in the United States is projected to surge by 700% between 2017 and 2060 [8], presenting a substantial health and economic burden if not addressed. In this review, childhood and adolescent T2D will be referred to as youth‐onset T2D.

Youth‐onset obesity and T2D are associated with an increased risk and earlier onset of serious long‐term health complications, such as retinopathy, nephropathy, cardiovascular disease, and metabolic dysfunction‐associated steatotic liver disease (MASLD) [9, 11–13]. Furthermore, increased exposure to stressful life events and adverse social determinants of health (SDOH), such as low household income, poor caregiver education, and food insecurity, are known to correlate with impaired quality of life and an elevated incidence of psychological problems, including depression, in patients with youth‐onset T2D [14, 15]. Youth‐onset T2D is associated with excess mortality such that patients with T2D aged ≤30 years have been shown to have a significant mortality excess and increased risk of death when compared to age‐matched patients with type 1 diabetes [16, 17]. The increasing incidence of youth‐onset T2D worldwide, combined with the heightened risk of serious health complications and death, highlights the urgent need for effective treatment strategies to combat this disease.

When compared with adult‐onset T2D, youth‐onset T2D has a more aggressive clinical course with rapid deterioration in β‐cell function necessitating initiation of insulin therapy. Patients with youth‐onset T2D can experience faster disease progression and earlier onset of severe complications, including microvascular and macrovascular complications compared with adult patients [18–20]. Furthermore, patients with youth‐onset T2D have almost a 50% lower peripheral and hepatic insulin sensitivity and are typically more insulin resistant than patients with adult‐onset T2D [21–23]. As such, established treatment options including lifestyle interventions and metformin may not demonstrate satisfactory glycemic control in patients with youth‐onset T2D.

Until recently, the major challenge in the management of youth‐onset T2D was the lack of treatment options; for nearly 20 years metformin and insulin were the only approved therapies available [24, 25]. Metformin was approved for use in patients aged 10 years or older by the Food and Drug Administration (FDA) in 2000 and prescribed as a first‐line oral antidiabetic therapy, with add‐on insulin therapy initiated when glycemic targets were not met [25]. Now, following the results from randomized clinical trials, new therapeutic agents across different drug classes have been approved for the treatment of youth‐onset T2D in the United States and Europe, including the injectable glucagon‐like peptide‐1 receptor agonists (GLP‐1 RAs) liraglutide, exenatide, and dulaglutide [26–28], and the orally active sodium–glucose cotransporter‐2 (SGLT‐2) inhibitors dapagliflozin and empagliflozin [29–31].

Despite multiple phase 3 studies assessing the efficacy and safety of new therapies in patients with youth‐onset T2D, challenges in patient recruitment make performing such trials extremely difficult [32]. Moreover, while GLP‐1 RAs and SGLT‐2 inhibitors have shown significant cardiovascular and renal benefits in adults with diabetes [33–38], there is a lack of large‐scale, long‐term phase 3 trials assessing these outcomes in young individuals with diabetes. As such, long‐term clinical data for GLP‐1 RAs and SGLT‐2 inhibitors in this population are limited, meaning clinicians often must rely on efficacy and safety data regarding cardiovascular and renal outcomes from adult patients. Discussing the cardiovascular and renal benefits of GLP‐1 RAs in adults with T2D is beyond the scope of this review; however, a recent meta‐analysis of randomized‐placebo‐controlled trials conducted by Rivera and colleagues reports cardiovascular and renal outcomes of GLP‐1 RAs in adults with and without T2D [39]. In this review, we summarize the current treatment landscape for youth‐onset T2D, examining the advantages and disadvantages of various treatments, and evaluate the clinical evidence for SGLT‐2 inhibitors in this patient population.

## 2. Current Treatment Landscape for the Management of Youth‐Onset T2D

The management of T2D in patients under 18 years of age is complex, requiring a combination of healthy lifestyle changes and pharmacologic intervention. Currently, international treatment guidelines from the American Diabetes Association (ADA), European Association for the Study of Diabetes (EASD), and International Society for Pediatric and Adolescent Diabetes (ISPAD) recommend metformin as the first‐line therapy for the management of youth‐onset T2D [24, 40, 41]. Add‐on insulin therapy is recommended in patients who are metabolically unstable (hemoglobin A1c [HbA1c] >8.5%) at diagnosis or for whom metformin alone does not provide glycemic control [20]. The Treatment Options for type 2 Diabetes in Adolescents and Youth (TODAY) and Restoring Insulin Secretion (RISE) trials highlighted the need for improved treatments in patients with youth‐onset T2D. The TODAY trial demonstrated that metformin only provided effective glycemic control in half of the participants, while in neither trial were metformin nor insulin able to effectively slow the accelerated decline in β‐cell function [42–46].

Between 2019 and 2023, three injectable GLP‐1 RAs were approved for use in children aged 10 years or older, either alone or in combination with metformin and/or insulin, by the FDA and European Medicines Agency (EMA). Once‐daily subcutaneous liraglutide was approved for the treatment of youth‐onset T2D by both the FDA and EMA in 2019 [26], followed by approval of once‐weekly subcutaneous exenatide by the FDA in 2021 and the EMA in 2022 [27]. More recently, once‐weekly subcutaneous dulaglutide was approved for use in youth‐onset T2D by the FDA in 2022 and the EMA in 2023 [28, 47, 48], and once‐weekly tirzepatide was approved by the FDA in 2025 [49, 50]. However, GLP‐1 RAs have been associated with an increased frequency of gastrointestinal side effects, including abdominal discomfort, nausea, vomiting, and diarrhea [20, 27, 28, 51]. Despite there currently being a lack of research into adherence to recently approved injectable therapies in youth‐onset T2D, there are well‐ known adherence issues associated with injectable insulin in young patients, thus highlighting the need for new and effective oral therapies in this population [25, 52, 53].

Given the known association between increasing childhood obesity and T2D, weight management treatments have an important role in the current treatment landscape in this indication. In the SURPASS‐PEDS trial, tirzepatide treatment resulted in significant reductions in body mass index (BMI) of adolescents aged 10 to <18 years with youth‐onset T2D. Patients treated with 5 mg tirzepatide for 30 weeks achieved a 7.4% reduction in BMI over 30 weeks (11.2% for 10 mg tirzepatide) versus 0.4% for placebo (*p* = 0.0001/*p*  < 0.0001 respectively) [49]. These doses were also associated with a significantly greater mean reduction in body weight of −6.8%/−10.5% (+0.4% for placebo) at Week 30. While not yet approved for the treatment of youth‐onset T2D, once‐weekly semaglutide has demonstrated clinically relevant decreases in BMI and body weight in adolescents (12 to <18 years of age) with obesity [54]. Semaglutide resulted in a mean change in BMI from baseline to Week 68 of −16.1% compared to + 0.6% for placebo, resulting in a statistically significant treatment difference of −16.7% (*p*  < 0.001). Furthermore, the treatment difference for semaglutide versus placebo in absolute change in body weight from baseline was −17.7 kg (a relative change of −17.4%) at Week 68 [54]. In 2019, oral semaglutide was the first orally active GLP‐1 RA approved for the treatment of adults with T2D by the FDA [55]. The ongoing PIONEER TEENs trial is currently assessing the efficacy and safety of oral semaglutide in patients aged 10–18 years with T2D [56].

The orally active SGLT‐2 inhibitors dapagliflozin and empagliflozin have also been approved for the treatment of youth‐onset T2D by both the EMA and FDA [29, 30, 57–60]. The currently available clinical efficacy and safety data for these SGLT‐2 inhibitors in the treatment of youth‐onset T2D are discussed in detail below.

## 3. The Role of SGLT‐2 Inhibitors in the Management of T2D

### 3.1. Mechanism of Action

**Figure 1:**
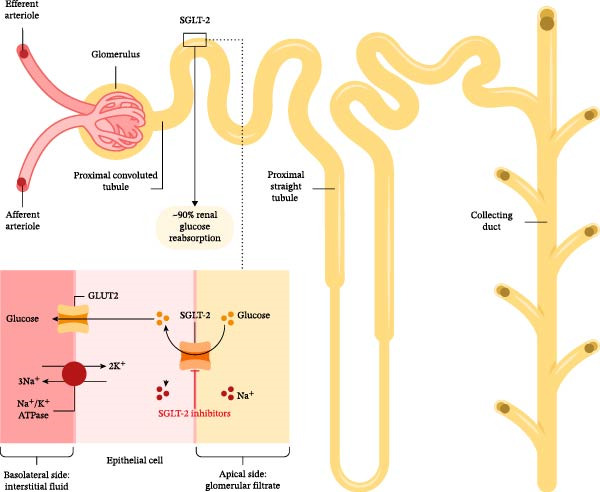
Mechanism of action of SGLT‐2 inhibitors. SGLT‐2 is predominantly expressed in the proximal convoluted tubule and is responsible for ~90% of renal glucose reabsorption [64]. SGLT‐2 inhibitors such as dapagliflozin and empagliflozin reduce glucose reabsorption in the kidneys and result in increased glucose excretion in the urine [63]. ATPase:‍ adenosine triphosphatase; GLUT2: glucose transporter 2; K^+^: potassium; Na^+^:‍ sodium; SGLT‐2: sodium–glucose cotransporter‐2.

The kidney is responsible for the filtration and reabsorption of circulating glucose [61]. In T2D, the threshold of renal glucose reabsorption is increased to a plasma glucose concentration of ≥200 mg/dL, leading to reduced urinary glucose excretion, which contributes to the hyperglycemia [62, 63]. These changes in renal glucose reabsorption are primarily attributed to the increased expression and/or activity of SGLT‐2, a high‐capacity transporter predominantly expressed in the proximal renal tubule and responsible for the majority of glucose reabsorption in the kidneys [64–66]. SGLT‐2 inhibitors are a new class of antihyperglycemic drugs that reduce reabsorption of glucose in the kidneys, lower the renal threshold for glucose, and increase glucose excretion in the urine by inhibiting the action of this transporter [63]. An overview of the mechanism of action of SGLT‐2 inhibitors is provided in Figure 1.

### 3.2. SGLT‐2 Inhibitors in the Management of T2D in Adults

SGLT‐2 inhibitors are well‐established in the treatment of adult‐onset T2D, with dapagliflozin and empagliflozin demonstrating efficacy in improving glycemic control in numerous clinical trials, both as monotherapies and add‐on therapies. In phase 2 b and 3 trials, mean HbA1c was reduced by −0.82% to −0.89% in adults treated with 10 mg dapagliflozin [67–69], and −0.50% to −0.82% in adults treated with 10 mg empagliflozin [70–73]. Furthermore, for both dapagliflozin and empagliflozin, patients achieved significant reductions in body weight [69, 71–74], and have also demonstrated improved cardiorenal outcomes in adult patients with and without T2D [33, 35, 75].

In patients with chronic kidney disease, with or without T2D, those treated with dapagliflozin had a reduced risk of the primary composite outcome of a sustained decline in the estimated glomerular filtration rate of at least 50%, end‐stage kidney disease, or death from renal or cardiovascular causes than those receiving placebo (9.2% versus 14.5%) [34]. Incident or worsening nephropathy has also been shown to be less frequent with empagliflozin treatment when compared to placebo in patients with T2D (12.7% versus 18.8%) [76].

As a result of these positive results from randomized clinical trials, dapagliflozin and empagliflozin have been approved by the EMA and FDA for the treatment of T2D with and without established cardiovascular disease, heart failure, and chronic kidney disease in adults [57–60]. The substantial and robust evidence of both cardiovascular and renal benefits of SGLT‐2 inhibitors in adults has further led to their recommendation in current clinical practice guidelines, especially for patients facing high risks of complications [77, 78]. However, given the aforementioned accelerated trajectory of early‐onset diabetes, established clinical efficacy from trials in adult‐onset T2D cannot necessarily be extrapolated to younger patients.

## 4. Clinical Efficacy and Safety of SGLT‐2 Inhibitors in the Treatment of Youth‐Onset T2D

Due to the difficulties in recruiting pediatric and adolescent patients in sufficiently high numbers, performing studies to assess the efficacy and safety of new treatments in youth‐onset T2D can prove extremely challenging [32]. Here, we have performed a targeted literature search to identify clinical trials examining the efficacy and safety of SGLT‐2 inhibitors in the treatment of youth‐onset T2D.

A search of PubMed was conducted on 12 December 2024 to identify clinical trials assessing the efficacy and safety of SGLT‐2 inhibitors in the treatment of youth‐onset T2D (patients <18 years of age), using the search term ((type 2 diabetes) OR (type 2 diabetes mellitus)) AND ((sodium–glucose co‐transporter‐2 inhibitor) OR (sodium–glucose cotransporter‐2 inhibitor) OR (SGLT‐2 inhibitor) OR (SGLT‐2 inhibitor) OR (empagliflozin) OR (dapagliflozin)) AND ((youth) OR (paediatric) OR (pediatric) OR (children) OR (adolescent) OR (childhood) OR (child) OR (newborn) OR (infant) OR (preschool)). Table 1 provides an overview of clinical trials of SGLT‐2 inhibitors in the treatment of youth‐onset T2D and their outcomes.

**Table 1:** Clinical efficacy of SGLT‐2 inhibitors in youth‐onset T2D.

Clinical trial number (trial name) [sponsor]	Location	Number of patients randomized	Background antidiabetic treatments [*n* (%)]	Intervention (administration route)	*N*	Age, years, mean (SD)	Primary outcome timepoint^a^	Adjusted mean HbA1c change from baseline (%)	Adjusted mean HbA1c change from baseline versus placebo (%)	Adjusted mean FPG change from baseline(mg/dL)	Adjusted mean FPG change from baseline versus placebo (mg/dL)
NCT02725593 (T2GO) [29] [AstraZeneca]	Multinational	72	Metformin: 37 (51.4) Insulin: 12 (16.7) Metformin + insulin: 23 (31.9)	Dapagliflozin 10 mg (oral, once‐daily)	39^b^	16.1 (3.3)	Week 24	−0.51^c^	**−1.13** ^**c**^ (**p** = 0.012)	−1.26^d,e^	−14.1^d^ (*p* = 0.340)
				Placebo (oral, once‐daily)	33^b^	16.2 (3.6)		0.62^c^	N/A	12.97^d,e^	N/A

NCT03429543 (DINAMO) [30] [Boehringer Ingelheim]	Multinational	158^f^	Metformin: 80 (51.0) Insulin: 5 (3.2) Metformin + insulin: 63 (40.1)	Empagliflozin 10 mg (oral, once‐daily)^g^	52	14.4 (1.9)	Week 26	−0.17	**−0.84**(**p** = 0.012)	−19.48 (*n* = 48)	−35.18
				Linagliptin 5 mg, (oral, once‐daily)	52	14.6 (1.9)		0.33	−0.34 (*p* = 0.29)	10.29 (*n* = 51)	−5.41
				Placebo (oral, once‐daily)	53	14.6 (1.8)		0.68	N/A	15.70 (*n* = 52)	N/A

NCT03199053 (T2NOW) [31] [AstraZeneca]	Multinational	245	Metformin: 126 (51.4) Insulin: 30 (12.2) Metformin + insulin: 89 (36.3)	Dapagliflozin 5 mg (oral, once‐daily)	81	14.4 (2.0)	Week 26	−0.62	**−1.03** (**p** < 0.001)	−10.3 (*n* = 81)	**−19.5** (**p** = 0.024)
				Placebo (dapagliflozin comparison, oral, once‐daily)^h^	76	14.7 (1.6)		0.41	N/A	9.2 (*n* = 74)	N/A
				Saxagliptin 2.5 mg (oral, once‐daily)	88	14.5 (1.8)		0.06	−0.44 (*p* = 0.078)	1.4 (*n* = 86)	−2.0
				Placebo (saxagliptin comparison; oral, once‐daily)^h^	76	14.7 (1.6)		0.50	N/A	3.4 (*n* = 74)	N/A

*Note:* Bold values indicate *p* < 0.05.

Abbreviations: FPG, fasting plasma glucose; HbA1c, hemoglobin A1c; N/A, nonapplicable; SD, standard deviation.

^a^Efficacy data presented here are those reported at the end of the double‐blind active treatment period.

^b^Intent‐to‐treat population; protocol‐compliant population consisted of 34 patients in the dapagliflozin arm and 26 patients in the placebo arm.

^c^Analysis of protocol‐compliant population.

^d^Analysis of intent‐to‐treat population.

^e^Data presented as mmol/L in original publication; dapagliflozin: adjusted mean FPG change from baseline = −0.07 mmol/L; placebo: adjusted mean FPG change from baseline = 0.72 mmol/L.

^f^157 patients received treatment.

^g^For empagliflozin, efficacy data were based on a pooled analysis for all participants on empagliflozin; participants in the empagliflozin group who failed to achieve HbA1c less than 7.0% (<53 mmol/mol) by Week 12 underwent a second double‐blinded randomization at Week 14 in a 1:1 ratio, either remaining on the empagliflozin 10 mg dose or increasing to the 25 mg dose.

^h^Separate analysis of covariance (ANCOVA) models were fit for dapagliflozin vs placebo and saxagliptin vs. placebo treatment effects.

### 4.1. Clinical Trials of SGLT‐2 Inhibitors in Youth‐Onset T2D

To date, there have been three pivotal phase 3 placebo‐controlled studies that have assessed the efficacy and safety of SGLT‐2 inhibitors in the treatment of youth‐onset T2D [29–31]. In two of these trials, DINAMO and T2NOW, another class of oral therapy, dipeptidyl peptidase‐4 [DPP‐4] inhibitors, were used as an active comparator [30, 31]. DPP‐4 inhibitors act by inhibiting the enzyme that inactivates incretin hormones, thereby increasing endogenous levels of active GLP‐1 and resulting in increased insulin secretion and reduced glucagon secretion [20]. Despite being well‐established in the treatment of adult‐onset T2D, DPP‐4 inhibitors have demonstrated limited or no efficacy in patients with youth‐onset T2D in the DINAMO, T2NOW, and other randomized clinical trials [30, 31, 79–81]. As such, DPP‐4 inhibitors have not been approved for the treatment of youth‐onset T2D and will not be discussed in detail in this review.

The T2GO (NCT02725593) trial assessed dapagliflozin or placebo as add‐on therapy in children and young adults aged 10–24 years of age with T2D receiving standard of care (metformin alone, insulin alone, or metformin plus insulin) [29]. Overall, 53 children (10–17 years of age) and 19 young adults (18–24 years of age) participated in the trial. Of those aged 10–17 years, 29/53 were randomly assigned to receive once‐daily oral dapagliflozin 10 mg, and 24/53 to placebo.

The DINAMO (NCT03429543) trial assessed empagliflozin or linagliptin (a DPP‐4 inhibitor) versus placebo in children 10–17 years of age with T2D previously treated with metformin, insulin, or metformin with insulin [30]. Of the 158 randomized patients, 157 patients received treatment: 52 were treated with once‐daily oral empagliflozin 10 mg, 52 were treated with linagliptin 5 mg, and 53 were treated with placebo.

More recently, the T2NOW (NCT03199053) trial assessed dapagliflozin or saxagliptin (a DPP‐4 inhibitor) versus placebo in patients 10–17 years of age with T2D receiving metformin, insulin, or metformin plus insulin [31]. The trial included 245 patients, of which 81 were randomized to receive once‐daily oral dapagliflozin 5 mg, 88 to saxagliptin 2.5 mg, and 76 to placebo.

### 4.2. Clinical Efficacy of SGLT‐2 Inhibitors

Dapagliflozin and empagliflozin have both demonstrated significant improvements in glycemic control in patients with youth‐onset T2D. In the T2GO phase 3 study, there was no significant difference between dapagliflozin and placebo for the primary endpoint of adjusted mean change from baseline in HbA1c at Week 24 in the intent‐to‐treat population. However, in a prespecified sensitivity analysis of protocol‐compliant participants, dapagliflozin demonstrated a clinically relevant decrease in HbA1c compared to placebo at Week 24 (adjusted mean change from baseline in HbA1c: −0.51% for dapagliflozin and +0.62% for placebo, resulting in a mean treatment difference of −1.13% [*p* = 0.012] in favor of dapagliflozin) [29]. In the T2NOW phase 3 study, dapagliflozin, but not saxagliptin, showed significant improvement in glycemic control compared with placebo. In patients 10–17 years of age with T2D, the adjusted mean change at Week 26 from baseline in HbA1c for dapagliflozin versus placebo was −1.03% (*p*  < 0.001), whereas for saxagliptin it was −0.44% (*p* = 0.078) [31]. Similarly, in the DINAMO phase 3 study, empagliflozin demonstrated a clinically relevant and statistically significant lowering in HbA1c compared to placebo, while linagliptin did not. Empagliflozin achieved the primary endpoint of adjusted mean HbA1c change from baseline at Week 26 versus placebo (−0.84%; *p* = 0.012), whereas linagliptin did not (−0.34%; *p* = 0.29) [30]. However, unlike the results reported in adults [71–74], neither dapagliflozin nor empagliflozin demonstrated notable reductions in body weight in patients under 18 years of age [29–31]. These results reflect the normal increases in growth and development that occur in individuals under 18 years of age, particularly in those who have undergone puberty. Despite this, given that youth‐onset obesity is a major risk factor for early‐onset T2D and the associated long‐term health complications, it is clinically important that clinicians also provide weight management strategies when treating T2D in patients under 18 years old.

Adjusted mean change in fasting plasma glucose (FPG) was a key secondary endpoint in the T2GO, DINAMO, and T2NOW phase 3 trials. Across all the studies, treatment with SGLT‐2 inhibitors resulted in numerical reductions in FPG when compared to placebo (Table 1) [29–31]. At Week 26 in the T2NOW trial, a difference in FPG of –19.5 mg/dL (*p* = 0.024) was reported between dapagliflozin and placebo [31]. Similarly, in the DINAMO trial, empagliflozin demonstrated a clinically relevant difference in FPG of –‍35.2 mg/dL at Week 26 versus placebo, whereas linagliptin did not (–5.4 mg/dL) [30]. Despite the T2GO trial reporting a difference in FPG of –‍14.1 mg/dL at Week 24 between the dapagliflozin and placebo groups, this was due to FPG values increasing in the placebo group, as by Week 24 FPG concentrations had returned to baseline values in the dapagliflozin group [29].

As discussed earlier, SGLT‐2 inhibitors have demonstrated positive cardiovascular and renal outcomes in adult patients with and without T2D [33–35, 75, 76]. However, it is currently not known whether these benefits will be replicated in patients under 18 years of age. The DOUBLE PRO‐TECT trial is currently recruiting patients with Alport syndrome aged 10–39 years to assess the benefits of dapagliflozin in young patients with chronic kidney disease [82, 83].

### 4.3. Safety and Tolerability of SGLT‐2 Inhibitors

Overall, dapagliflozin and empagliflozin have been demonstrated to be well tolerated in patients with youth‐onset T2D after 52 weeks of treatment, with a safety profile generally comparable to studies in adult patients. Hypoglycemia was a commonly reported adverse event for patients with youth‐onset T2D receiving SGLT‐2 inhibitors over 52 weeks in the T2GO, DINAMO, and T2NOW phase 3 trials (dapagliflozin:‍ 33.3% [T2GO], 29.6% [T2NOW]; empagliflozin: 19.3% [DINAMO]) [29–31]. Hypoglycemia was, in general, less frequently reported in adult patients with T2D treated with dapagliflozin or empagliflozin, occurring in ~5% of patients [67–69, 71, 73, 74, 84]. Haring and colleagues did, however, report hypoglycemia in up to 16.1% of adult patients treated with empagliflozin as an add‐on therapy to metformin plus sulfonylurea [72]. Across the T2GO and T2NOW trials, severe hypoglycemia occurred in three (7.7%; T2GO) and four (4.9%; T2NOW) patients receiving dapagliflozin, whereas no cases of severe hypoglycemia requiring assistance were reported following empagliflozin treatment in the DINAMO trial [29–31]. Furthermore, the occurrence of hypoglycemic events in patients receiving dapagliflozin was more common in those participants also treated with insulin. In T2GO, 10 out of the 13 patients that experienced hypoglycemia were also receiving insulin, while in T2NOW, all patients who experienced severe hypoglycemia were also being treated with insulin. Incidence rates of genital infections in youth‐onset T2D have been demonstrated to be comparable to adults following treatment with SGLT‐2 inhibitors, occurring in ~5%–10% of patients in both populations [30, 31, 67–69, 71–73, 84]. However, in the T2GO trial, one female patient had to discontinue dapagliflozin treatment due to a genital infection [29].

In a real‐world clinical setting euglycemic diabetic ketoacidosis is a common concern with SGLT‐2 inhibitor treatment. A recent systematic review and network meta‐analysis of randomized controlled clinical trials assessed the risk of diabetic ketoacidosis with the use of active antidiabetic drugs in adults with T2D. The study by Yang and colleagues revealed that SGLT‐2 inhibitors and other antidiabetic drugs were not associated with an increased risk of diabetic ketoacidosis when compared to placebo [85]. Similarly, across the T2GO, DINAMO, and T2NOW phase 3 trials, there was only one reported case of diabetic ketoacidosis associated with SGLT‐2 inhibitor treatment [29–31]. At Week 52, 1 (1.2%) patient receiving dapagliflozin in the T2NOW study reported diabetic ketoacidosis, with the number of reported cases of diabetic ketoacidosis similarly low in both the saxagliptin (0) and placebo (1 [1.3%]) treatment arms. The reported incidence of diabetic ketoacidosis in patients with youth‐onset T2D following treatment with dapagliflozin and empagliflozin is comparable with studies in adult patients [33, 34, 75, 76]. However, these data were collected in the controlled environment of randomized clinical trials, where patients are carefully monitored, which is not necessarily the case in real‐world clinical practice. As such, clinicians still need to be cautious when prescribing SGLT‐2 inhibitors for patients with T2D with a history of diabetic ketoacidosis and counsel their patients regarding the risks associated with diabetic euglycemic ketoacidosis. The accurate clinical distinction between early‐onset T2D and diabetes with underlying genetic causes (maturity‐onset diabetes of the young [MODY] and latent autoimmune diabetes [LADA]) is of particular importance, with reports of heightened risks of diabetic ketoacidosis and acute kidney injury reported in some patients with these conditions treated with SGLT‐2 inhibitors following misdiagnosis of early‐onset T2D [86, 87].

Currently, studies into the long‐term effects of SGLT‐2 inhibitor treatment in patients under 18 years of age are limited. Of the phase 3 studies that have evaluated the efficacy and safety of dapagliflozin and empagliflozin in youth‐onset T2D, only the T2NOW study included a 52‐week nontreatment follow‐up period that assessed measures of growth and maturity, Tanner staging, markers of bone health, and safety following the withdrawal of dapagliflozin or saxagliptin in this patient population [88]. The follow‐up period demonstrated that prior treatment with dapagliflozin or saxagliptin for 52 weeks did not raise any safety concerns relating to height, weight, BMI, Tanner staging, growth and maturation markers, bone biomarkers, or adverse events up to 1 year after treatment. However, further studies are required to fully understand the long‐term efficacy and safety of SGLT‐2 inhibitors in patients with youth‐onset T2D and given the chronic nature of the condition, will give valuable insight into whether the efficacy and safety signals observed to date in this population are sustained with continued use.

As a result of these positive results from the T2GO, DINAMO, and T2NOW phase 3 clinical trials, dapagliflozin and empagliflozin have both been approved for use in patients with youth‐onset T2D. In 2022, dapagliflozin was the first orally active treatment since metformin to be approved by the EMA for use in patients aged 10 years or older [29, 57]. The FDA subsequently approved dapagliflozin for use in youth‐onset T2D in 2024 [59]. Empagliflozin was approved for the treatment of youth‐onset T2D in patients aged 10 years and above by both the FDA and EMA in 2023 [30, 58, 60]. In December 2024, the FDA approved canagliflozin for use in patients aged 10 years or older with T2D [89]; however, at the time of publication, canagliflozin is not approved for use in youth‐onset T2D by the EMA [90]. At the time of writing, the results of the completed study assessing the efficacy and safety of canagliflozin in patients aged 10–17 years with T2D (NCT03170518) have not yet been published, and as such have not been discussed in this review [91].

## 5. Current Challenges in the Management of Youth‐Onset T2D

As described above, substantial improvements have been made in the treatment landscape for youth‐onset T2D over recent years, with the approval of multiple new therapeutic options across a range of drug classes and several ongoing trials (Table 2) [26–31]. As such, paucity of treatments is no longer an acute challenge in the management of youth‐onset T2D.

**Table 2:** Recruiting clinical trials to assess the efficacy and safety of pharmacological therapies in youth‐onset T2D.

Clinical trial number (Trial name) [Sponsor]	Study title	Location	Inclusion criteria	Intervention (administration route)	Ages eligible for study	Primary endpoints	Study duration
NCT05990374 [92] [Nanjing First Hospital, Nanjing Medical University]	Therapeutic effects and effects on body Fat of GLP‐1 receptor agonists in patients with T2D for 1–4 Years	China	Diagnosed with T2D	Dulaglutide (subcutaneous injections, once‐weekly) Semaglutide (subcutaneous injections, once‐weekly) Loseenatide (subcutaneous injections, once‐weekly) Tirzepatide (subcutaneous injections, once‐weekly) Elbenatide (subcutaneous injections, once‐weekly) Placebo (subcutaneous injections, once‐weekly)	Child, adult, older adult	Changes of blood glucose fluctuation after treatment	1–4 years

NCT05067621 [93] [Yale University]	Semaglutide effects in obese youth with prediabetes/new onset T2D and nonalcoholic fatty liver disease	United states	Diagnosed with pre‐impaired glucose tolerance (2 h glucose ≥130–≤200 mg/dL post‐OGTT) OR impaired glucose tolerance (2 h glucose ≥140–<200 mg/dL post‐OGTT OR HbA1c ≥5.7–‍<6.5%) OR new onset T2D (≤24 months duration, 2 h glucose >200, and HbA1c >6.5%–10%)	Semaglutide 2.4 mg (subcutaneous injection, once‐weekly) Placebo (subcutaneous injection, once‐weekly)	Child, adult (10–<21 years of age)	Change in oral disposition index Change in protein density fat fraction	6 months followed by a wash‐out period of 3 months

NCT06739122 [94] (AWARD‐PEDS PLUS) [Eli Lilly and Company]	A Study of Dulaglutide (LY2189265) 3.0 mg and 4.5 mg in pediatric participants with T2D mellitus	United states	T2D treated with diet and exercise and metformin and/or basal insulin	Dulaglutide 3.0 mg (subcutaneous injection) Dulaglutide 4.5 mg (subcutaneous injection)	Child (10–17 years of age)	Number of participants with ≥1 serious adverse events considered by the investigator to be related to study drug	8 months

NCT04881799 [95] [University of Minnesota]	Phentermine/Topiramate in adolescents with T2D and obesity	United States	T2D and obesity (BMI ≥95^th^ percentile for age and sex)	Phentermine 3.75–15 mg/Topiramate 23–92 mg (orally, once‐daily)^a^ Placebo (orally, once‐daily)	Child, Adult (12–≤20 years of age)	Change from baseline in BMI	6 months placebo‐controlled period, followed by a 6‐month open label extension

NCT05008276 [96] (PANTHER study) [Petter Bjornstad, Seattle Children’s Hospital]	Puberty, diabetes, and the kidneys, when eustress becomes distress	United states	Youth with overweight/obesity (BMI ≥85^th^ percentile) and/or newly diagnosed T2D and elevated HbA1c (HbA1c ≥6%)	Aminohippurate sodium injection 20% Iohexol injection 300 mg/mL Dextran 40 sodium injection 0.9%	Child (8–14 years of age)	Effective renal plasma flow Glomerular filtration rate	2 years

*Note:* Searches of ClinicalTrials.gov were conducted on 2 May 2025 to identify recruiting clinical trials to assess the efficacy and safety of pharmacological therapies in patients with youth‐onset T2D, using the search term Type 2 Diabetes AND Not yet recruiting, recruiting studies AND Child (birth–17).

Abbreviations: BMI, body mass index; HbA1c, hemoglobin A1c; OGTT, oral glucose tolerance test; T2D, type 2 diabetes.

^a^Doses to be titrated from an initial dose of phentermine 3.75 mg/topiramate 23 mg to a maximum dose of phentermine 15 mg/topiramate 92 mg.

Despite the approval of multiple new therapies following phase 3 trials in youth‐onset T2D, the well‐known challenges in patient recruitment in pediatric and adolescent studies are still an issue [32]. Therefore, despite patients under 18 years of age typically displaying a more aggressive disease phenotype than adult T2D patients, there is currently not enough clinical data in the former group to support the same protective effects demonstrated by new drug classes in adult patients with T2D.

Youth‐onset T2D is a global health concern that disproportionately affects nonwhite, ethnically diverse, and socioeconomically disadvantaged subgroups [3–5, 7]. The TODAY trial demonstrated that a patient’s race or ethnic subgroup had a significant effect on treatment outcome, with metformin monotherapy less effective in non‐Hispanic Blacks (an overall treatment failure rate of 66.2%) when compared to non‐Hispanic whites (an overall treatment failure rate of 44.9%, *p* = 0.01) or Hispanics (an overall treatment failure rate of 44.0%, *p*  < 0.001) [42]. For optimal management of T2D, clinicians therefore need to consider differing response rates to T2D treatments across racial subgroups. However, these racial subgroups are often underrepresented in phase 3 trials, with many studies not representative of the populations primarily impacted by T2D.

SDOH are nonmedical factors that influence health outcomes, including the conditions in which people are born, age, educated, work and live [97]. A patient’s SDOH can present a major challenge in the management of youth‐onset T2D, impacting both their access to medication and adherence to treatment. Furthermore, in the United States, inequalities in insurance can limit a patient’s access to certain treatments. As such, SDOH can greatly affect the likelihood of a patient achieving treatment goals.

## 6. Conclusion

The phase 3 trials of SGLT‐2 inhibitors in youth‐onset T2D (T2GO, DINAMO, and T2NOW) have demonstrated the clinical efficacy and safety of dapagliflozin and empagliflozin in the treatment of this patient population. Treatment with dapagliflozin and empagliflozin resulted in statistically significant and clinically relevant improvements in glycemic control compared with placebo. However, in contrast to previous studies of SGLT‐2 inhibitors in adults with T2D, treatment with dapagliflozin and empagliflozin did not result in significant reductions in body weight in patients with youth‐onset T2D. Overall, SGLT‐2 inhibitors were well tolerated in this patient population, and the safety profile was consistent with previous studies in adult patients. However, hypoglycemia was a commonly reported adverse event in patients with youth‐onset T2D, occurring more frequently when compared to adult patients. These trials indicate that the orally administered SGLT‐2 inhibitors offer an effective treatment option for patients with youth‐onset T2D. Given the known adherence issues with injectable therapies in this patient population, oral treatments may have an important role in achieving optimal management of this disease.

Currently, the evidence for the use of SGLT‐2 inhibitors in the treatment of youth‐onset T2D is generated in clinical trials and may not be reflective of the wider youth‐onset T2D patient population. Furthermore, despite the recent publication of the 52‐week nontreatment follow‐up period of T2NOW, there is currently limited evidence on the long‐term efficacy and safety of SGLT‐2 inhibitors in this patient population. Moreover, it remains unknown whether there are differences in response rates to SGLT‐2 inhibitors across racial subgroups. Further follow‐up studies in addition to real‐world evidence are necessary to examine the long‐term potential of SGLT‐2 inhibitors in addressing the unmet need for optimal management of youth‐onset T2D and its serious comorbidities.

## Author Contributions

Substantial contributions to study conception and design and to analysis and interpretation of the data, drafting the article or revising it critically for important intellectual content, and final approval of the version of the article to be published: Silva Arslanian, Timothy Barrett, and Naim Shehadeh.

## Funding

This article was funded by AstraZeneca. Support for third‐party writing assistance for this article, provided by David Morgan, PhD (Costello Medical, UK), was funded by AstraZeneca in accordance with Good Publication Practice (Grant GPP 2022) guidelines (https://www.ismpp.org/gpp-2022).

## Ethics Statement

This article reports data from previously conducted studies and does not contain any new studies with human participants or animals performed by any of the authors.

## Conflicts of Interest

Silva Arslanian: DMC chair for two pediatric trials for Eli Lilly; consultant for Eli Lilly and Novo Nordisk; clinical trials by Novo Nordisk. Timothy Barrett: supported by UK NIHR as a Senior Investigator. Naim Shehadeh: global medical director for AstraZeneca T2NOW (NCT03199053) trial; consultant for AstraZeneca, Boehringer Ingelheim, Eli Lilly, and Novo Nordisk.

## Data Availability

This article is a literature review, and no novel data were generated.
